# Conservative Treatment of Invasive Cervical Resorption Using Mineral Trioxide Aggregate (MTA) and Fiber-Reinforced Composite: A Case Report

**DOI:** 10.7759/cureus.85487

**Published:** 2025-06-06

**Authors:** Shweta R Rathi, Pratima Shenoi, Chetana S Makade, Shriya Shahu, Teena Oommen

**Affiliations:** 1 Department of Conservative Dentistry and Endodontics, Ranjeet Deshmukh Dental College and Research Centre, Nagpur, IND; 2 Department of Conservative Dentistry and Endodontics, Vidya Shikshan Prasarak Mandal (VSPM) Dental College and Hospital, Nagpur, IND; 3 Department of Oral and Maxillofacial Surgery, Ranjeet Deshmukh Dental College and Research Centre, Nagpur, IND

**Keywords:** cbct, external cervical resorption, fiber-reinforced post and core, invasive cervical resorption, mineral trioxide aggregate (mta), ribbond, root resorption

## Abstract

Invasive cervical resorption (ICR) is not only a rare but also an aggressive dental condition that can further progress, leading to significant structural damage and tooth loss if not diagnosed and managed early. The following case report delves into the successful conservative treatment of a maxillary lateral incisor affected by ICR in a 33-year-old man with a history of dental trauma. Routine clinical and radiographic evaluation, along with cone-beam computed tomography (CBCT), confirmed Heithersay class III resorption with subcrestal involvement. The treatment involved root canal therapy, surgical debridement, and defect restoration with mineral trioxide aggregate (MTA), followed by Ribbond fiber-reinforced (Ribbond, Inc., Seattle, WA) composite for structural reinforcement. A follow-up of six months postoperatively showed no recurrence of resorption, and the patient remained asymptomatic, demonstrating the effectiveness of a multidisciplinary approach. This case highlights the importance of CBCT-guided diagnosis, biocompatible materials, and minimally invasive techniques in the management of invasive cervical resorption.

## Introduction

Root resorption refers to the continuous degradation and loss of dental hard tissues. While it is a natural and necessary physiological occurrence in primary teeth, when it affects permanent teeth, it is considered a pathological condition that can ultimately lead to tooth loss.

Root resorption is classified based on its origin, i.e., internal and external. Internal resorption occurs inside the pulpal chamber and progresses outward, whereas external resorption originates from within the periodontium, progressing further to affect the external surface of the tooth. External resorption can be further categorized into four distinct types: external cervical resorption (ECR), external inflammatory resorption, external replacement resorption, and external surface resorption. Each type presents unique etiological factors, clinical manifestations, and treatment considerations, necessitating accurate diagnosis and appropriate management to thwart further irreversible damage to the affected tooth [1].

A unique form of external root resorption, external cervical resorption, primarily initiates in the cervical third region of the root, which may progress in an apical, coronal, or bidirectional manner. In the initial stages, the dental pulp typically remains uninvolved; however, as the resorptive process advances, it may eventually lead to root perforation and pulpal involvement. The prevalence of invasive cervical resorption (ICR) has been reported to range from 0.02% to 0.08% in various epidemiological studies [2].

The pathogenesis of ECR remains a subject of debate, as multiple hypotheses are proposed. The most widely accepted theory suggests that structural defects or damage to the cemental layer beneath the epithelial attachment exposes the root surface to the activity of the osteoclasts, resulting in progressive dentinal resorption.

A comprehensive descriptive analysis conducted by Lin et al. indicated that a history of parafunctional habits (23.2%), dental trauma (28.5%), and orthodontic treatment (45.7%) were among the most frequently reported etiological factors [3].

Early detection, the complete eradication of the resorptive process, and appropriate restorative intervention are crucial for ensuring optimal clinical outcomes in cases of external cervical resorption (ECR). The European Society of Endodontology (ESE) Position Statements delineates various treatment strategies, including external and internal approaches, intentional replantation, periodic assessment, and, when necessary, extraction [4].

This case report details the conservative management involving root canal therapy, surgery, and the restoration of a maxillary lateral incisor exhibiting invasive cervical resorption, demonstrating a successful clinical outcome.

## Case presentation

A 33-year-old male patient was referred to the Department of Conservative Dentistry and Endodontics with a complaint of pinkish-black discoloration of the maxillary left lateral incisor (tooth number 22). The patient noticed a gradual color change over 3-4 months. Medical history revealed a history of trauma to the tooth four years back, and the patient did not seek any treatment for the same.

**Figure 1:**
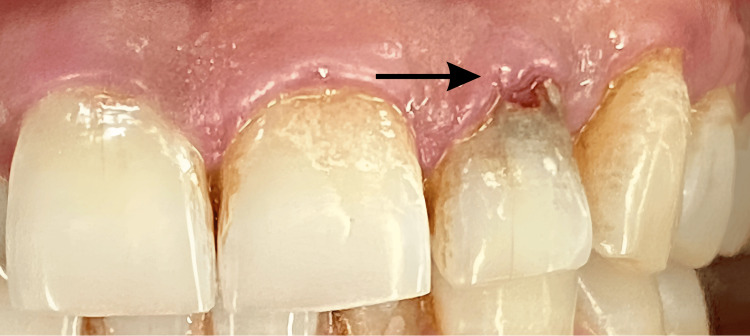
Preoperative clinical picture wrt tooth number 22. The black arrow indicating the pinkish-black discoloration on the labial surface wrt tooth number 22. wrt: with respect to

Upon clinical examination, discoloration was observed on the labial surface of tooth number 22 in the cervical region. On probing, a “catch” was detected with subsequent bleeding. The tooth showed no sensitivity to percussion and did not respond to either electric or thermal pulp tests, suggesting a non-vital tooth (Figure 1).

**Figure 2:**
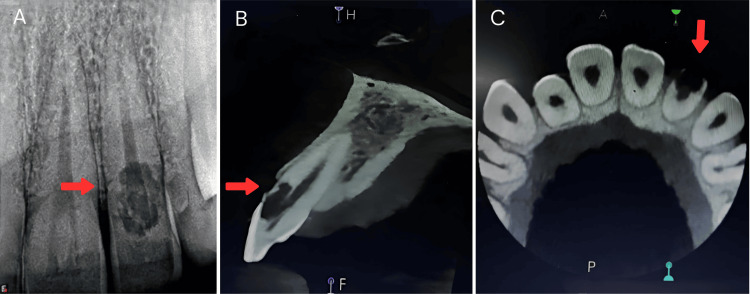
(A) Preoperative periapical radiograph; (B) CBCT image in sagittal section corresponding to tooth number 22; (C) CBCT axial section view of tooth number 22. (A) The red arrow depicts an irregular radiolucency with a mottled appearance. (B and C) The red arrow shows the extent of the lesion in the sagittal and axial sections of CBCT, respectively. CBCT: cone-beam computed tomography

Radiographic assessment reported an irregular radiolucency with a mottled appearance, involving enamel, dentin, and pulp and extending into the coronal third region of the root. The lesion was classified according to Heithersay’s two-dimensional (2D) classification (1999) as class III (indicating a deeper invasion of dentin, extending into the coronal third of the root) (Figure 2A), and cone-beam computed tomography (CBCT) examination further confirmed radiolucency involving enamel, dentin, and pulp, with the thinning of the buccal cortical plate [5]. According to Patel and Dawood’s 3D classification (2007), the lesion was categorized as height class II (subcrestal) [6]. The circumferential spread was classified as B (90-180 degrees), with probable pulpal involvement (P-type) (Figure 2B, 2C).

A differential diagnosis was established to distinguish between various cervical lesions commonly encountered in clinical practice. The table summarizes the distinguishing features of cervical caries, abfraction, and erosion, focusing on their etiology and clinical/radiographic findings. This classification was essential in accurately identifying the lesion type in the present case, thereby guiding appropriate treatment planning (Table 1).

**Table 1:** Differential diagnosis for external cervical resorption.

Lesion	Etiology	Findings
Cervical caries	Bacterial metabolism	Ill-defined radiolucency and normal pulp tests
Abfraction	Functional and parafunctional activities, including biting, chewing, clenching, and bruxism	V-shaped lesion with a smooth surface, usually located at the gumline
Erosion	Acidic food and beverages	A smooth, often concave saucer-shaped lesion on the tooth surface

After taking informed consent from the patient, the root canal procedure was initiated using local anesthesia (lignocaine hydrochloride with adrenaline 1:100000) and rubber dam isolation. Access opening was made from the palatal side, and working length was accurately determined using an apex locator (Root ZX Mini Apex Locator, J Morita, Kyoto, Japan) in conjunction with radiographic confirmation. After achieving the glide path using a #10K hand file, biomechanical preparation was completed (shaping with S1 and S2 and apical preparation up to F3) using rotary files (ProTaper Gold, Dentsply Sirona, Charlotte, NC) with copious intermittent irrigation with normal saline, 17% ethylenediaminetetraacetic acid (EDTA), and 2.5% sodium hypochlorite. A calcium hydroxide intracanal medicament (RC Cal Calcium Hydroxide Paste, Prime Dental, Thane, India) was placed for 14 days to aid in disinfection, and the cavity was sealed with temporary restoration (Cavit™ G Temporary Dental Filling Material, 3M, St. Paul, MN). The patient was recalled after two weeks.

**Figure 3:**
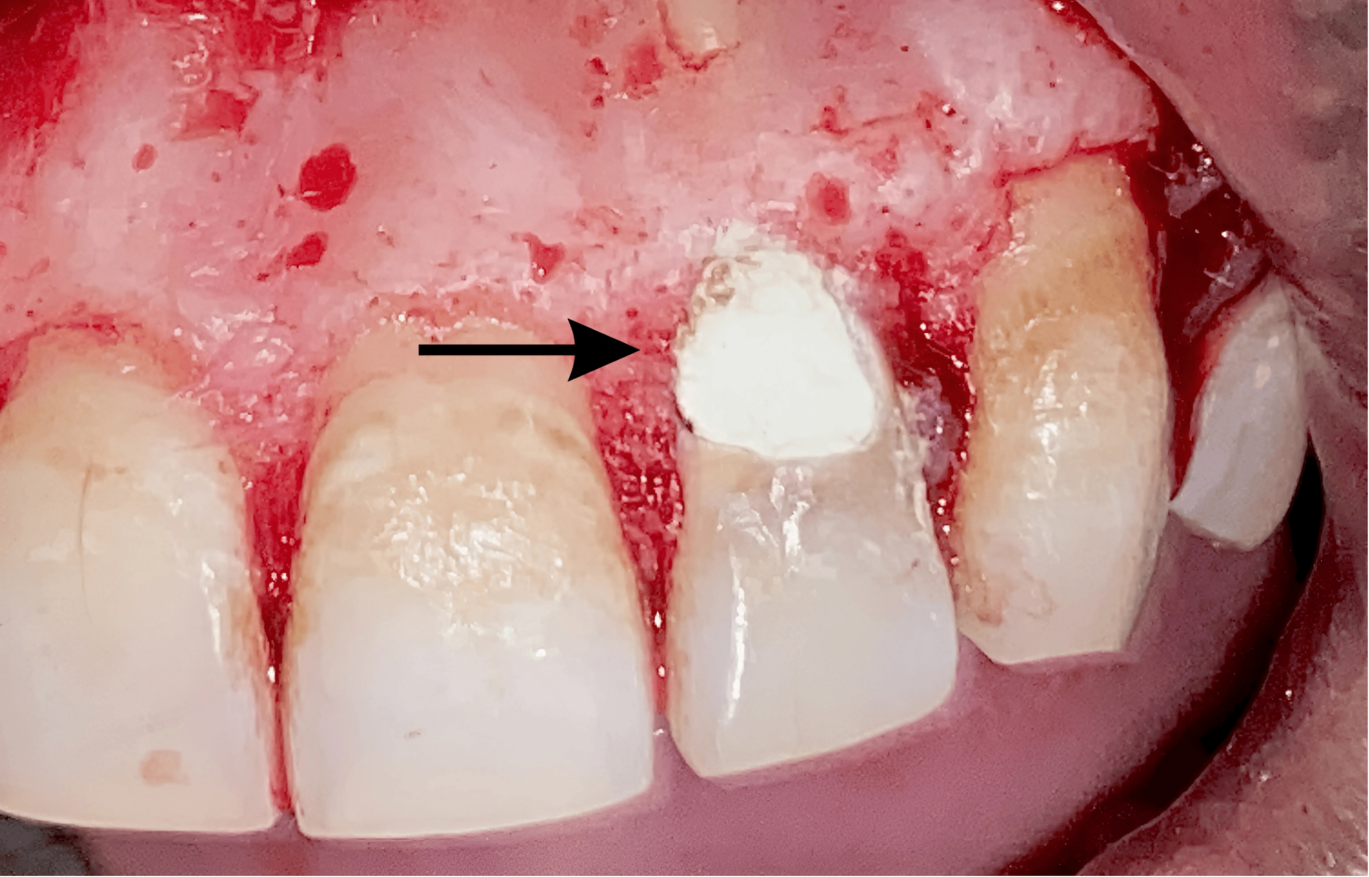
Clinical image showing surgical exposure, the debridement of granulomatous tissue, and the placement of mineral trioxide aggregate (MTA) over the resorptive defect wrt tooth number 22. The black arrow indicates the MTA material placed at the site of the defect after surgical exposure wrt tooth number 22. wrt: with respect to

As the resorption defect was perforating into the root canal space, a surgical approach was undertaken to repair the defect. The access cavity was temporarily sealed using a gutta-percha point (size 300.06 taper), avoiding the obstruction of the root canal with filling material. A full-thickness labial mucoperiosteal flap was reflected under local anesthesia. Upon exposure, granulomatous tissue was observed extending along the labial surface to the cervical third of the crown. The resorptive tissue was gently excised with a curette, and bleeding was controlled. The site was then isolated, and a cotton pellet moistened with 5% sodium hypochlorite (Sodium Hypochlorite 5.25%, Prime Dental, Thane, India) was placed to allow effective penetration into areas of the resorptive defect that were difficult to access [7,8]. Following thorough disinfection, a biocompatible material, mineral trioxide aggregate (MTA) (ProRoot MTA, Dentsply Sirona, Charlotte, NC), was mixed as per the manufacturer’s instructions and was placed over the resorptive defect. Finally, the flap was repositioned and secured using vertical mattress sutures with 5-0 vicryl sutures (Figure 3).

**Figure 4:**
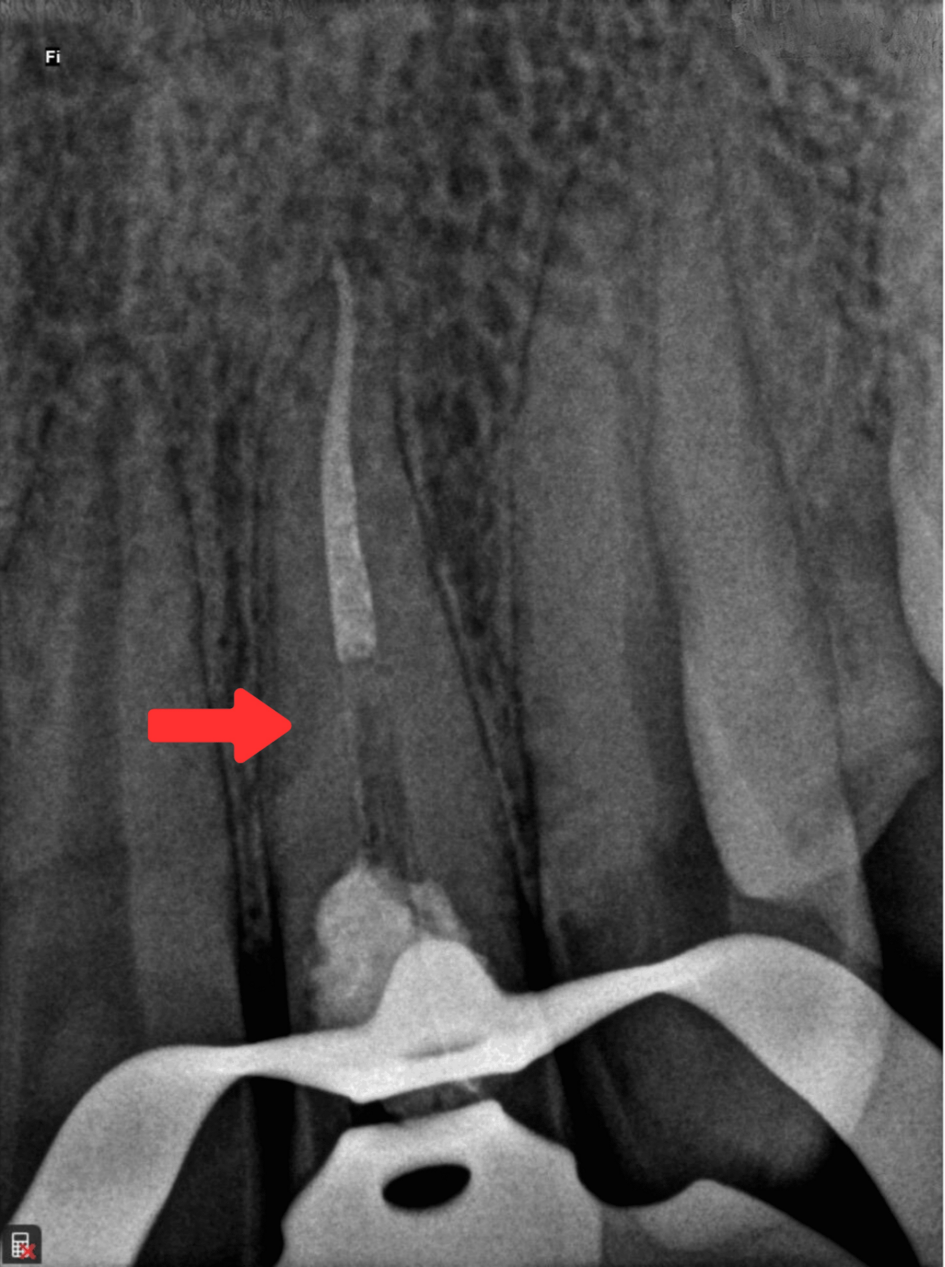
Postoperative radiograph showing the sectional obturation along with placement of Ribbond fibers. The arrow shows the Ribbond fibers placed in the canal after making post space preparation.

After one week, the patient was recalled, and root canal treatment was completed using the sectional obturation technique. The post space was then prepared using Peeso reamer number 4 (Peeso Reamers 32 mm, Mani, Utsunomiya, Japan). The required length of Ribbond fiber (Ribbond® bondable reinforcement ribbon 4 mm) (Ribbond, Inc., Seattle, WA) was measured, considering twice the depth of the post space and the estimated core height. The canal was etched with 37% phosphoric acid, rinsed, and gently air-dried. A bonding agent (3M ESPE, St. Paul, MN,) was applied, air-dried, and light-cured according to the manufacturer’s instructions. Following this, a dual-cure resin cement (RelyX ARC, 3M ESPE, St. Paul, MN) was mixed and applied to the canal. Ribbond fibers were folded into a V-shape and tightly condensed in the canal with an endodontic plugger, followed by light curing for 30 seconds (Figure 4).

**Figure 5:**
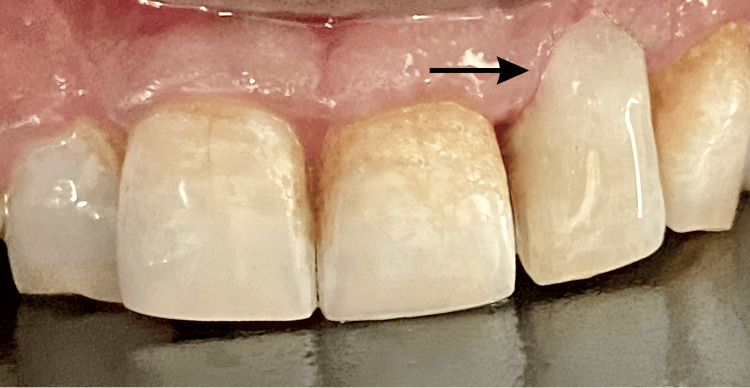
Postoperative clinical photograph with tooth number 22. The arrow depicts the post-endodontic restoration using microhybrid composite restorative material wrt tooth number 22. wrt: with respect to

Post-endodontic restoration was done using microhybrid composite restorative material (Te-Econom Plus Composite, Ivoclar Vivadent, Schaan, Liechtenstein) (Figure 5).

**Figure 6:**
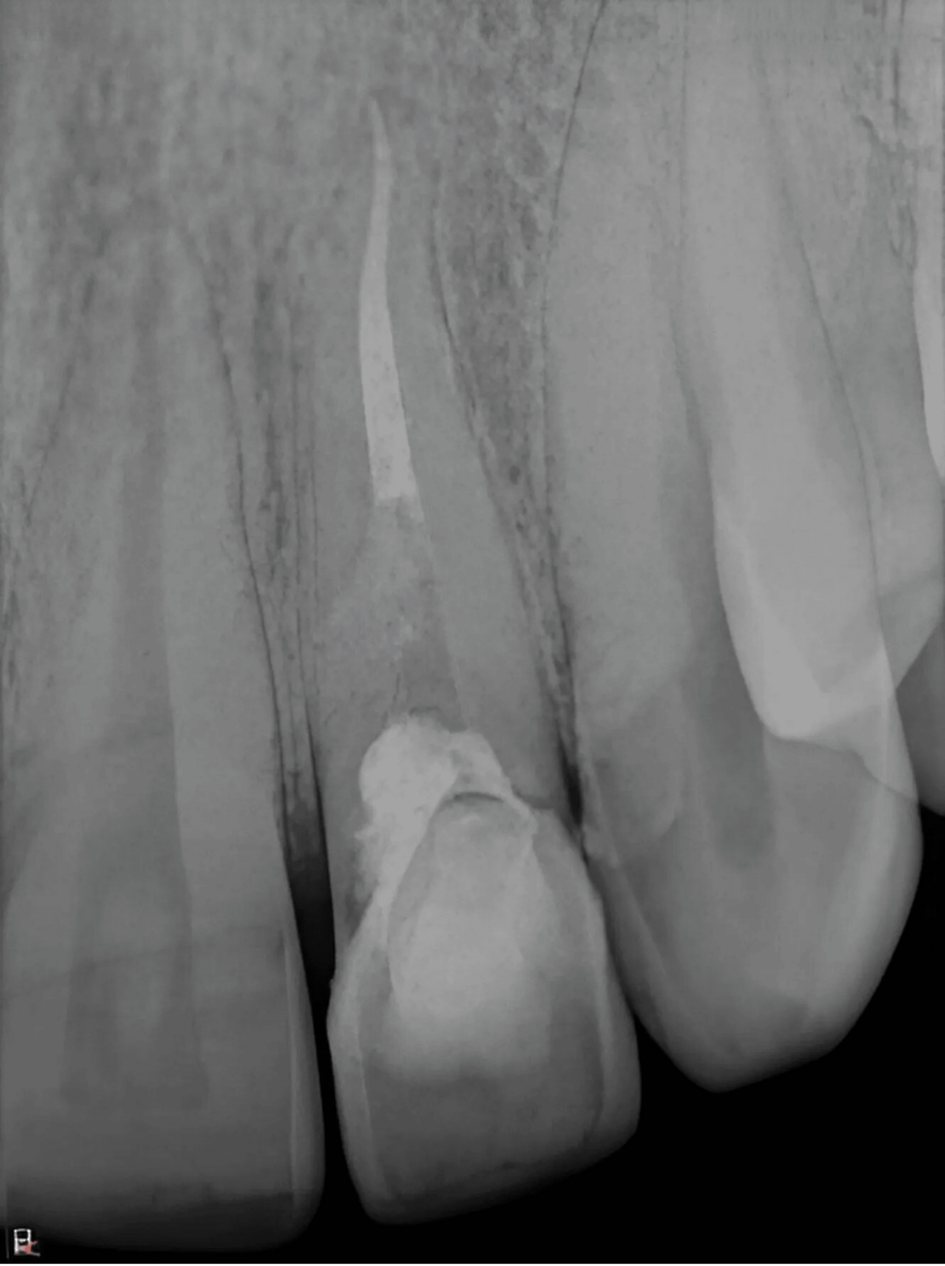
Six-month follow-up postoperative radiograph with no periapical pathology wrt tooth number 22.

A six-month follow-up radiograph shows successful healing with no periapical pathology and healthy surrounding bones (Figure 6). Clinically, the treated tooth remained asymptomatic, with no pathological periodontal findings.

## Discussion

Dental trauma is commonly identified as a significant predisposing factor for invasive cervical resorption, as it compromises the integrity of the periodontal ligament, cementum, and adjacent alveolar bone. Previous studies have identified maxillary incisors, canines, and first molars as the most commonly affected teeth in cases of ICR. Studies have suggested that factors such as orthodontic treatment, internal bleaching, dental surgery, bruxism, and extensive restorations may contribute to the initiation and progression of ICR [9].

A key diagnostic challenge in this case was differentiating between external cervical resorption (ECR) and internal root resorption (IRR), as IRR with cervical perforation may present a similar radiographic appearance to ECR. Accurate differentiation is essential for appropriate treatment planning. Conventional two-dimensional radiography often lacks the resolution needed to distinguish between these conditions effectively. Therefore, cone-beam computed tomography (CBCT) plays a crucial role in diagnosis. In this case, preoperative CBCT facilitated the precise localization and assessment of the resorptive lesion, providing critical insights into its nature and extent. Based on the radiographic and clinical findings, the resorptive defect was classified as Heithersay’s class III ECR.

The present case report demonstrates a favorable prognosis, as the resorptive lesion was confined near the coronal pulp without extending fully into the radicular dentin, thereby enhancing the long-term success of the treatment. The patient presented with progressive discoloration of the maxillary left lateral incisor following a traumatic injury sustained four years prior.

Various treatment modalities have been reported for the management of ECR. External treatment approaches for managing resorptive lesions include non-surgical external access, orthodontic extrusion, intentional reimplantation, and surgical flap reflection. An internal treatment approach involves accessing the resorptive tissue nonsurgically and removing it chemically and/or mechanically through root canal treatment, and an amalgamation of the two approaches, integrating both external and internal techniques, may be used for more comprehensive management. In the present case, a surgical flap was raised to treat the subgingival resorptive defect. According to a systematic review by Bardini et al., external access with a surgical flap was reported to be the most frequently used treatment modality for external root resorption (61%) [4].

The primary objectives in treating a tooth with resorption are halting the active resorption, repairing the defect, and preserving the tooth’s functionality. Bioactive bioceramic materials, such as mineral trioxide aggregate (MTA), are preferred for repairing resorptive defects due to their favorable tissue response and outstanding biocompatibility. It has also been reported to promote dentinogenesis and facilitate dentin bridge formation. Studies have consistently demonstrated its superior ability to induce dentin repair compared to calcium hydroxide, yielding faster reparative dentin formation and enhanced structural integrity [1,10]. Hence, MTA was used as a choice of material in the present case.

Post-endodontic restoration was done using Ribbond. An important advantage of Ribbond, a fiber-reinforced composite material developed by Dr. David N. Rudo, is its ability to enhance the strength and longevity of restorative treatments while preserving natural tooth structure. Unlike conventional post-and-core systems that require extensive root canal modification, which eventually weakens the root dentin, Ribbond offers a minimally invasive alternative, reducing the risk of further tooth weakening. Its structural design, consisting of 215 densely interwoven fibers, functions as an intrinsic crack-arresting mechanism by redirecting crack propagation and dissipating mechanical stress. Additionally, its adaptability allows it to adapt to irregular canal morphologies, making it particularly effective in cases where standard dowel systems may be unsuitable. Beyond its structural benefits, it improves the performance of composite resin restorations by enhancing their flexural strength and fracture resistance. Its integration with composite resins is further optimized by a “gas plasma” surface treatment, which reduces fiber tension and strengthens chemical adhesion, improving bond integrity. Furthermore, Ribbond’s translucent fibers blend seamlessly with restorative materials, mimicking the optical properties of the surrounding composite resin. This feature allows for natural light transmission through restorations and crowns, resulting in superior aesthetic and functional outcomes in restorative dentistry [11].

Posttreatment, the patient remained asymptomatic, and radiographic evaluations during the follow-up period of six months revealed no evidence of peri-radicular pathology. While the current case showed a positive outcome, additional long-term studies with extended follow-up periods are required to thoroughly evaluate the efficacy and clinical reliability of MTA and Ribbond in the treatment of external cervical resorption.

## Conclusions

The effective management of invasive cervical resorption (ICR) necessitates an accurate diagnosis and a meticulously planned treatment strategy. Surgical intervention provides direct access to the resorptive defect, facilitating comprehensive debridement and optimal sealing with MTA, known to promote periodontal healing and serve as a durable barrier against further resorption. Additionally, the incorporation of Ribbond fiber-reinforced composite enhances the structural integrity of the root and supports coronal restoration, ensuring long-term functional and aesthetic outcomes. This approach underscores the critical role of precise surgical techniques and advanced restorative materials in achieving predictable and successful management of ICR cases.
